# Prevention of chemotherapy-induced nausea and vomiting with acupuncture: A protocol for systematic review and meta-analysis

**DOI:** 10.1097/MD.0000000000018828

**Published:** 2020-01-17

**Authors:** Ting-Ting Ma, Tao Zhang, Gan-Lin Zhang, Cun-Fang Dai, Bo-Ran Zhang, Xiao-Min Wang, Lin-Peng Wang

**Affiliations:** a Beijing University of Chinese Medicine; b Oncology Department; c Acupuncture and Moxibustion Department, Beijing Hospital of Traditional Chinese Medicine, Capital Medical University, Beijing, China.

**Keywords:** acupuncture, chemotherapy-induced nausea and vomiting, effect, safety

## Abstract

**Background:**

Nausea and vomiting are the most common complications following chemotherapy and usually lead to decreased quality of life. Acupuncture therapy is an effective method for chemotherapy-induced nausea and vomiting (CINV), the effects and safety have been observed by many clinicians and demonstrated in a systematic review, which was included in the Cochrane Library in 2014. After several years, new studies have occurred and an updated systematic evaluation is needed. This protocol describes a method for performing a systematic review and meta-analysis to further evaluate the beneficial effects and safety of acupuncture for CINV.

**Methods:**

A searching strategy will be carried out mainly in eight databases in English and Chinese, Cochrane Central Register of Controlled Trials, PubMed, Embase, China National Knowledge Infrastructure, the Chinese Scientific Journal Database, the Wanfang database, China Doctoral Dissertations Full-text Database, and China Master's Theses Full-text Database. Only randomized controlled trials related to acupuncture for CINV will be included to enhance the effectiveness. The effective percentage will be used as primary outcome. Changes in the symptoms of nausea and vomiting, like severity, duration, and frequency as well as quality of life will be assessed as secondary outcome. Side effects and adverse events will be used as safety evaluations. To ensure the quality of the systematic evaluation, study selection, data extraction, and quality assessment will be independently performed by 2 authors, and the third author will deal with any disagreement. The Review Manager V.5.3.3 s will be used to perform the data synthesis and subgroup analysis.

**Results:**

There are additional studies, further explanations and more subgroup analyses compared with the previous systematic analysis to determine the effects and safety of acupuncture for CINV.

**Conclusion:**

The result of this systematic review may offer clinicians stronger evidence to assist patient in relieving CINV.

**Ethics and dissemination:**

There is no need to acquire ethical approval for individuals come from literatures instead of recruiting directly. The findings of this review will be reported in peer-reviewed publications and/or presented at relevant conferences

**Trial registration number:**

CRD42016045223.

## 1 Introduction

With high incidence of cancer worldwide, the burden of the disease is increasing.^[1–3]^ Chemotherapy is currently an important therapy for treating cancer. However, nearly most of patients suffer from chemotherapy-induced nausea and vomiting (CINV),^[4–7]^ which can lead to a series of harmful consequences,^[8]^ such as electrolyte disturbances, dehydration, and lack of appetite. Furthermore, more severe complications, such as bleeding due to injury to the digestive tract mucosa, infection, and even death, may occur. Therefore, CINV not only influences the quality of life (QoL) but also physiological-psychological functions, and even result in large costs and bad compliance.^[8]^

Although the mechanisms underlying CINV are not clear enough,^[9]^ to date, commonly prescribed medicines^[10]^ for nausea and vomiting are: 5-hydroxytryptamine-3 serotonin receptor antagonists, tachykinin NK1 receptor antagonist, steroids, histamine receptor-blocking agents, dopamine receptor-blocking agents. However, these medicines for nausea and vomiting do not have sufficient curative effects^[11]^; moreover, increasing the dosage to achieve better efficacy may result in other side effects,^[12]^ such as headache, dizziness, and constipation. Conversely, acupuncture treatment for nausea and vomiting has advantages that include efficacy^[13–15]^ low cost, convenience, and safety.

Historically, acupuncture has played an important role in protecting the health of Chinese people for more than 2000 years and is increasingly being recognized and is gaining popularity in some Western countries.^[16,17]^ In 2009, the Cochrane Library system evaluated 67 types of diseases, of which 7 kinds including CINV,^[18]^ were confirmed to have been effectively treated with acupuncture. Acupuncture may have antiemetic effects mediated via regulation of visceral nerves, transmitters, and affection of gastrointestinal rhythm.^[19–21]^

Accordingly, this report presents a protocol for a systematic review of the prevention of CINV with acupuncture to ensure its curative effects and safety.

## 2 Objectives

The objective of this study is to update the systematic review^[22]^ and further evaluate effects and safety of acupuncture on CINV. Moreover, we aim to explore whether acupuncture can improve the QoL and compliance of chemotherapy patients.

## 3 Methods

The methods of this systematic review will be guided by the preferred reporting items for systematic reviews and meta-analyses statement.^[23]^

### 3.1 Inclusion criteria

#### 3.1.1 Types of studies

Randomized controlled trials related to CINV will be included for meta-analysis in this systematic review.

#### 3.1.2 Participants

Participants affected by CINV will be included in the meta-analysis regardless of their race and sex. The age range will be set to 18 to 80 years for better compliance and responses. Participants with nausea and vomiting secondary to motion sickness or nervous system disease will be excluded. Additionally, patients with mental disorders will be excluded. The specific criteria are as follows:

##### 3.1.2.1 Inclusion criteria

(1) Diagnosed with cancer with an indication for chemotherapy and an expected survival time of more than 3 months.
(2) Age between 18 and 80.
(3) Either gender.
(4) Good physical condition, with a Kamofsky score >60.
(5) Absence of digestive system diseases.

##### 3.1.2.2 Exclusion criteria

(1) Sever cardiac insufficiency, hepatic or kidney failure, or serious uncontrolled infection.
(2) Central nervous system pathology involving tumors, digestive tract obstructions with hydrothorax or ascites or any other disease that may cause nausea and vomiting.
(3) Metal disorders or any other diseases influence compliance.

#### 3.1.3 Interventions

Cancer is a unique disease, which requires that patients in both treatment and control group undergo basic treatment. In the treatment group, the intervention will be acupuncture or acupuncture with antiemetic drugs, acupuncture can be of any form as mentioned in the search strategy. Under normal conditions, patients in control group receive common drugs that prevent CINV nausea and vomiting instead of being left completely untreated. In the control group, no active treatment or sham acupuncture for the purpose of blinding are encouraged.

#### 3.1.4 Types of outcome measures

The primary outcome will be the effective percentage in the treatment. The secondary outcomes will include the appetite grading may refer to simplified nutritional appetite questionnaire^[24]^, the frequency and dosage of remedial antiemetic medications, the QoL rating scale score, and any other clinical assessments.

All reported side effects and adverse events will be included as safety outcomes. The common side effects of acupuncture, such as local pain, hematomas, and fainting, as well as complications, such as electrolyte disturbances, deprivation of body fluid, lack of appetite, bleeding resulting from digestive tract mucosal injuries, infection, and death, will also be recorded and evaluated respectively.

### 3.2 Search methods

#### 3.2.1 Electronic searches

We will search for relevant studies in the following databases: Cochrane Central Register of Controlled Trials, PubMed, Embase, China National Knowledge Infrastructure, the Chinese Scientific Journal Database, the Wanfang database, China Doctoral Dissertations Full-text Database, and China Master's Theses Full-text Database. Additionally, we will search for as much information as possible from the following sources: the reference lists of the included studies, unpublished conference proceedings, and ongoing trials, which will be identified using the World Health Organization International Clinical Trials Registry Platform (http://apps.who.int/trialsearch/) and Current Controlled Trials (http://www.controlled-trials.com).

#### 3.2.2 Search strategy

The following terms will be used for the search:

A. Search strategy to identify “nausea and vomiting”:
  (1) Nausea
  (2) Vomiting
  (3) Emesis
  (4) Sick
  (5) Naupathia
  (6) Lack of appetite
  (7) Or/1–6
B. Search strategy to identify “chemotherapy”:
  (8) Chemotherapy
  (9) Chemotherapeutics
  (10) 10 Or/8–9
C. Search strategy to identify acupuncture interventions:
  (11) Acupuncture
  (12) Electroacupuncture
  (13) Body acupuncture
  (14) Auricular acupuncture
  (15) Ear acupuncture
  (16) Scalp acupuncture
  (17) Intradermal needle
  (18) Fire needle
  (19) Elongated needle
  (20) Warm needle
  (21) Dry needle
  (22) Or/11-21

### 3.3 Data collection and analysis

#### 3.3.1 Selection of studies

All the authors in our team will be trained regarding the purpose and process of the review. The selection work will require at least 3 independent authors, 2 of whom will perform the study selection, and the third author will adjudicate any disagreement throughout the process. Specifically, 2 authors will first select studies by reading titles, abstracts, and if necessary, the full texts. Then, to ensure the authenticity of the collected studies, the authors may search the related published protocols or contact either the first author or the corresponding author.

#### 3.3.2 Data extraction and management

Data extraction will also require 3 independent authors. Two will conduct the extraction and cross-checking work from the included studies, and the third author will adjudicate any disagreements during the process. All extracted data will be put into an extraction form which is developed according to the recommendations of the Cochrane Handbook. The extraction form will consist of general information items: the author, work location, publication date, journal, participants, characteristics of the interventions, outcomes, randomization, allocation concealment, incomplete data, blinding, selective reporting, adverse events, follow-up, sources of funds, and conflicts of interest.

#### 3.3.3 Assessment of the risk of bias

The 2 independent authors mentioned above will first evaluate the risk of bias separately and then cross-check their findings. The third author will help resolve any potential disagreements. We will grade risk of bias as “high risk,” “low risk,” and “unclear risk” of bias using the “Risk of bias” tool from the Cochrane Handbook (V.5.1.0). We will assess the following types of bias:

(1) selection bias, including random sequence generation and allocation concealment;
(2) performance bias, including blinding of the investigators, participants, and operators;
(3) detection bias, including the blinding of the outcome assessment, and in particular, the blinding of the data assessor;
(4) attrition bias, which refers to incomplete data/differential dropout;
(5) reporting bias, including selective reporting, whether or not all data are reported; and
(6) other bias, including conflicts of interest, such as sponsorships from companies, and deficiencies in the follow-up involving some of the participants not being contacted again.

#### 3.3.4 Measurements

The results of the studies will be integrated and presented as risk ratios with 95% confidence intervals (95% CI) for dichotomous data and as mean differences or standardized mean difference with 95% CIs for continuous data.

#### 3.3.5 Missing data

Because studies are not regulated by a particular fixed format, the data presented in each article may not be consistent. In such cases, the authors may contact either the first or corresponding author to ask for the required data by via telephone or email.

#### 3.3.6 Assessment of heterogeneity

The Mantel–Haenszel *χ*^2^ test for heterogeneity will be used to calculate statistical heterogeneity. Significant heterogeneity will be identified by *P* > .1, or *I*^2^ < 50%. As described in the Cochrane Handbook, there are 4 categories of heterogeneity as determined by the *P*-value: Little or no heterogeneity, *P* < 40%; Moderate heterogeneity, 30% < *P* < 60%; Substantial heterogeneity, 50% < *P* < 90%; Considerable heterogeneity, 75% < *P* < 100%. At the same time, we will use fixed-effect model if *I*^2^ < 50% or *P* > .10, otherwise, we may consider random effect model and descriptive analysis.

#### 3.3.7 Assessment of reporting biases

If 10 or more trials are selected for the meta-analysis, we will analyze the potential publication bias by generating funnel plots. Quantitative analysis will be carried out by using Egger test.^[25]^

#### 3.3.8 Data synthesis

Meta-analysis will be performed with Review Manager (V.5.3) statistical software provided by Cochrane Collaboration. A fixed effect model will be carried out if there is little significant heterogeneity among the trials, or a random effect model will be established.

#### 3.3.9 Subgroup analysis

A single meta-analysis is difficult to analyze if included studies contain obvious differences, analysis of the data in a single meta-analysis will be difficult. In such cases, subgroup analyses will be performed, and the studies will be distinguished according to the following:

(1) type of acupuncture therapy,
(2) treatment timing (eg, before or after the chemotherapy),
(3) grade of nausea and vomiting,
(4) types of chemotherapy drugs, and
(5) types of symptoms including acute vomiting, delayed vomiting, and anticipatory vomiting.

#### 3.3.10 Sensitivity analysis

To evaluate the robustness and stability of the pooled outcomes, sensitivity analysis is needed by sequentially eliding each trial. When a low-quality study is identified, we may perform the meta-analysis with and without that study and subsequently compare the 2 results to determine whether the low-quality study should be included. The final result will depend on the sample size, missing data, risk of bias and quality of methods of each study.

#### 3.3.11 Evidence quality

Grading of recommendations assessment, development and evaluation^[26]^ will be used to evaluate the quality of evidence as 4 levels: very low, low, moderate, and high.

## 4 Discussion

Acupuncture have exact effects on CINV, a systematic review of acupuncture-point stimulation for CINV was published over 10 years ago, and although that review was revised in 2014 by the same team,^[22]^ the studies included in the update did not include those studies since 2006. However, due to the rapid pace of acupuncture development, several new studies testing and verifying the curative effect of acupuncture on CINV were performed during this period. Therefore, updating the systematic review is necessary.

The updated review will differ from the former one^[30,31]^ by the following. ① Only 11 studies were included in the older systematic review, we will search for and include additional studies to obtain more data for the new analysis. ② The previous review only reported a significant reduction in the proportion of patients who experienced acute vomiting and a marginally significant change in the proportion of patients with reduced acute nausea severity, whereas the new systematic review will attempt to further explain these findings and report more aspects. ③ For delayed vomiting or nausea, no outcomes were significantly improved by noninvasive stimulation techniques. Accordingly, we will include additional studies to enable a reasonable subgroup analysis to demonstrate a more definite conclusion. ④ Study selection, data extraction, and quality assessment will be independently performed by 2 reviewers, and any disagreements will be adjudicated by a third reviewer. This process will improve the quality of the analysis and the review. ⑤ Notable dissimilarities may have been caused by the “dose,” 1 point was stimulated until Deqi was elicited. Therefore, in our review, “dose” will be considered one of the main influential factors in the evaluation of the curative effects of the treatments. ⑥ For the evaluation of safety, our review will search for and collect additional data from recent studies. Furthermore, we will focus on how to prevent the occurrence of CINV, the treatment of cancer patients with acupuncture before chemotherapy rather than the use of acupuncture as a method to relieve existing pain.

Despite these efforts, limitations in this systematic review will still exist. For example, we will only search English and Chinese databases. Additionally, as there are different types of acupuncture interventions, as mentioned above, and different types of nausea and vomiting, such as acute, delayed, and postoperative nausea and vomiting, subgroup analyses will be needed. In the present, we cannot quantify the stimulation of acupuncture.

## Author contributions

**T-T M, T Z, X-M W** and **L-P W** conceived of and designed this study. **T-T M** and **T Z** developed the search strategy and wrote the first draft of the protocol. **C-F D** and **B-R Z** will independently perform the specific work, such as screening the potential studies, performing the data extraction, assessing the risk of bias, entering the data into RevMan, and completing the data synthesis. X**-M W** will make the final decision when any disagreement arises. **T Z** will ensure that no errors occur during the review. **G-L Z, L-P W,** and **X-M W** revised the protocol. All authors have read and approved the final manuscript.
